# Effect of Ankle-Foot Orthoses in Pediatric Patients with Hereditary Motor-Sensory Neuropathy: A Case Series Study

**DOI:** 10.3390/children10091529

**Published:** 2023-09-09

**Authors:** Corrado Borghi, Silvia Sassi, Daniela Pandarese, Samuele Messori, Silvia Faccioli

**Affiliations:** 1 Children Rehabilitation Unit—UDGEE, Santa Maria Nuova Hospital, Azienda USL, IRCCS di Reggio Emilia, 42123 Reggio Emilia, Italy; silvia.sassi@ausl.re.it (S.S.); daniela.pandarese@ausl.re.it (D.P.); samuele.messori98@gmail.com (S.M.); silvia.faccioli@ausl.re.it (S.F.); 2 PhD Program in Clinical and Experimental Medicine, Department of Biomedical, Metabolic and Neural Sciences, University of Modena and Reggio Emilia, 41125 Modena, Italy

**Keywords:** Orthotic Devices, Gait Analysis, Rehabilitation, Hereditary Sensory And Motor Neuropathy, Postural Balance

## Abstract

(1) Aims: to evaluate the effect on gait performance and standing stability of ankle-foot orthoses (AFO) in pediatric patients with hereditary motor-sensory neuropathy (HMSN). (2) Methods: a retrospective case-series study including three adolescents (S1, S2, S3, mean age 14 years) with HMSN. The subjects were evaluated barefoot, with carbon AFO (Botter) and with solid AFO (SAFO) by means of: gait analysis, stabilometry and gait functional tests (10 Meter Walk Test, 2 Minute Walk Test). Finally, the CSD-OPUS questionnaire was administered to the assess satisfaction and impact of the orthoses on life quality. (3) Results: orthoses improved gait and stability performance. Botter allowed greater ankle movement than SAFO and provided greater push-off power. This, combined with the carbon elastic energy return, might explain better performances in the 2MWT, with a larger distance traveled compared to SAFO for both S1 (110 m vs. 72 m) and S2 (170 m vs. 155 m) and, compared to barefoot walking, also for S3 (211 m vs. 160 m), for which SAFO analysis was not available. Both orthoses improved performance at the stabilometric analysis. The CSD-OPUS questionnaire showed a significantly higher level of satisfaction with Botter for the subjects (S1, S2) who completed the comparison. (4) Conclusions: Both orthoses improved gait and standing, though Botter proved to be better tolerated and more effective in improving gait endurance.

## 1. Introduction

The Hereditary sensory-motor neuropathies (HMSN), also known as Charcot-Marie-Tooth, have a prevalence of about 1 in every 2500 people [1]. Common clinical features are muscle atrophy, weakness, fatigue, balance instability, skeletal deformities, hyporeflexia or areflexia [2,3].

The first muscles to weaken and atrophy are the intrinsic muscles of the foot (which generate foot deformities), followed by the dorsal-flexors (which induce foot drop) and plantar-flexors (push-off reduction). This deficit leads to a reduction in stride length and speed [4,5]. As the disease progresses, the most proximal muscles are also affected [6]. In case of the quadriceps femoris deficit, knee hyperextension may arise as a stabilization strategy during the stance phase of the gait cycle [7,8,9].

Ounpuu et al. (2013) reported that pelvic elevation and increased hip (and knee) flexion during swing were a compensatory strategy for foot drop, while the increased external rotation of the hip was used to cope with the possible internal rotation of the foot [10]. Increased hip abduction was recurrent among both children and adults to widen the support base and obtain stability during walking. Ferrarin et al. (2012) recorded an early plantar-flexor peak in mid-stance (“vaulting” strategy) to favor the clearance of the contralateral foot [11]. Muscle weakness, in adjunct to a deficit of proprioception and skeletal deformities, may also lead to postural instability issues (Bigelow and Jackson, 2014, performed a battery of tests including force-plate posturography, the Mini Balance Evaluation Systems Test and the Timed Up and Go [12]).

The treatments adopted to counteract the symptoms and slow down their progression are the use of assistive devices and orthoses, physiotherapy and functional orthopedic surgery. All these tools aim at improving the quality of life of patients [2,13,14].

Ankle-foot-orthoses (AFO) are the most frequently used orthotic devices: they provide stability and support by containing the peripheral deficit, offering dynamic walking, improving balance and postural control, and decreasing energy expenditure [15,16,17].

In particular, carbon AFOs are specifically designed to store energy during the stance and increase ankle power during push-off. Their application to eight patients with HMSN of variable ages was studied by Dufek et al. (2014), who found an increase in ankle peak power, in power absorption at the hip and in spatio-temporal parameters, compared to barefoot walking [18]. On the contrary, Bigelow and Jackson (2014) did not identify any increase in these parameters in 12 patients with non-hereditary neuropathy [12]. Nonetheless, they found an improvement in postural control, by applying “Toe-Off” AFO (a nonarticulated, rigid carbon composite ankle-foot orthosis with an anterior shank support). Recently, Waterval et al. (2019) compared carbon posterior spring orthosis (DLS-AFO) with different stiffness levels in 37 patients of heterogeneous age (>18 years; mean age 56.9 ± 15.5 years) and with different neuromuscular disorders (only 16 were affected by HMSN), but with a common non-spastic weakness of the plantar-flexor muscles (typical alteration also in HMSN) [19]. In this study, the gait with carbon AFOs was compared to the gait with shoes only. The authors found that more flexible orthoses permit a lower energy expenditure during walking. They hypothesized that optimal stiffness to reduce energy cost is a compromise between a stiffness sufficient to normalize the ankle and knee kinematics and a flexibility that allows the generation of ankle power. No relevant effects were recorded on ankle power generation, in disagreement with the findings of Dufek et al. (2014) [18,19].

Therefore, there is no agreement in the literature regarding the effect of carbon AFOs in patients with neuropathies, since improvements in push-off power, spatio-temporal parameters and postural stability have not been found consistently [12,18,19]. Moreover, there is a lack of studies regarding the use of carbon AFOs in the pediatric population with this disease: to the authors’ knowledge, no studies were performed on the pediatric population and only one specifically considered patients with HSMN (only eight adults [18]). Concerning the effect of carbon AFOs on postural stability in patients with neuropathies, it was addressed in only one study, in 12 non-HMSN adult patients [12]. Furthermore, in all these works, no direct comparisons were made with AFOs of different materials, in order to verify whether the generally more expensive carbon is worth the additional costs. The aim of this case series study was to describe the benefits of two types of AFO (dynamic and solid) in the gait and postural stability of pediatric patients with HMSN. Both types of AFO should increase stability by providing support to the ankle, whose muscles are weakened. A dynamic AFO is expected to achieve more ankle propulsion and improve gait performance compared to both barefoot and solid AFO.

## 2. Case Series Presentation

Three adolescents were examined in this retrospective case series study (Table 1). Since childhood, they attended the Children Rehabilitation Unit—UDGEE, Santa Maria Nuova Hospital, AUSL-IRCCS of Reggio Emilia—for regular follow-ups. The research was carried out in accordance with the World Medical Association’s code of Ethics (Declaration of Helsinki, 1967) and the standards of the local Ethics Committee. The subjects’ parents provided written informed consent.

All subjects presented a chronic motor sensory polyneuropathy. They all had normal cognitive levels, and none had undergone surgery in the 6 months prior to the evaluations. Only one of them (Subject 2) underwent surgery four years prior to the present study, at the age of 10. Cerebral and spine Magnetic Resonance Imaging (MRI) were negative.

Subject 1 (S1) presented a predominant axonal involvement, though demyelination was also present, as demonstrated by repeated electroneurography and electromyography (at 3 and 11 years). Next, generation Sequencing (NGS) panel tests conducted so far resulted negative and further exams are ongoing. Clinical onset was at 3 years, with delayed and abnormal walking. Predisposed AFO were initially introduced, then substituted by customized SAFO at 4 years. Finally, carbon AFO were provided at 14 years, with an angle of >90° at the ankle level, to accept hyperextension compensation. S1 presented a bilateral pes cavus, with talus valgus on the right and dynamic varus on the left during stance.

Subject 2 (S2) also had genetic tests in the process, in particular, a IGHMBP2 variant was found which might have a pathogenetic role. Alterations in the PMP22, GDAP1 and MFN2 genes were excluded. She acquired independent walking at 18 months and persisted, asymptomatic, for 4 years. Afterwards, falls and gait pattern alterations started, then a predisposed AFO was introduced (PEROMED^®^, Roma, Italy). In the last 6 years, upper limb’s deficits also appeared and diagnostic assessments began. S2 developed a severe right-foot deformity in terms of equinus-cavus-varosupination. Then orthopedic surgery was performed at 10 years on the right leg, including gastrocnemius aponeurotomy, extension osteotomy of first digit’s metatarsal, tibialis posterior to tibialis anterior transfer, flexor hallucis and digitorum longus tenotomy and plantar aponeurotomy. After surgery, the right foot reached a good alignment at the clinical exam, but varosupination was still present when barefoot during the stance. Conversely, mild valgopronation was observed on the left. To preserve the alignment obtained by means of surgery, bilateral SAFO was introduced, which was finally substituted by carbon AFO.

Subject 3 (S3) presented CMT2s based on pathogenetic variants of the IGHMBP2 gene. He acquired independent walking at 18 months. Cerebral MRI at 3 years showed an isolated left peritrigonal heterotopic nodule, which remained asymptomatic and stable at the subsequent MRI at 13 years. At the time of the study, he had been using carbon AFO for the last 2 years, instead of the previously customized SAFO. He presented mild cavus on the left and severe cavus-varosupination on the right foot.

## 3. Interventions

All subjects were provided with AFOs to improve gait.

S1 and S2 wore bilateral SAFO, but when renewal was needed because of overuse, they changed to Botter, which appeared to be more suitable to clinicians. On the contrary, S3 had been using Botter over the last 2 years.

**Figure 1:**
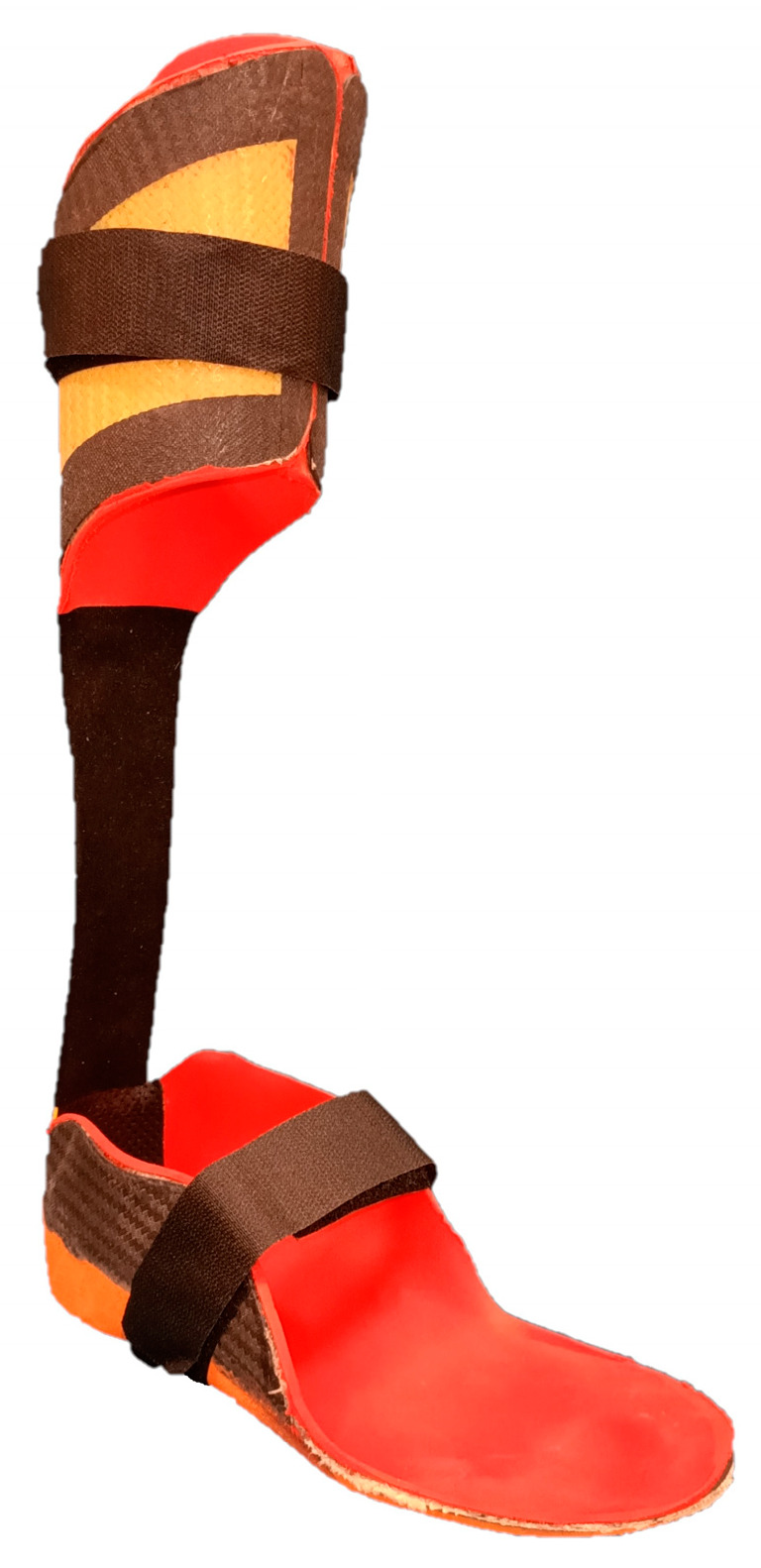
Example of AFO Botter used by the patients.

The Botter orthosis was custom molded. It had a base with a rear part adapted to the heel of the shoe and a forefoot pointing upwards (Figure 1). Posteriorly, it presented a crossbow made up of carbon and aramid fibers. The desired result, in terms of resistance, elasticity and dynamic response, was obtained thanks to the particular arrangement and layering of the fibers of which the brace was made. Stiffness of the forefoot and of the crossbow, and ankle angle at rest, were individually tailored based on the patient’s weight and height and on the experience of the technicians.

**Figure 2:**
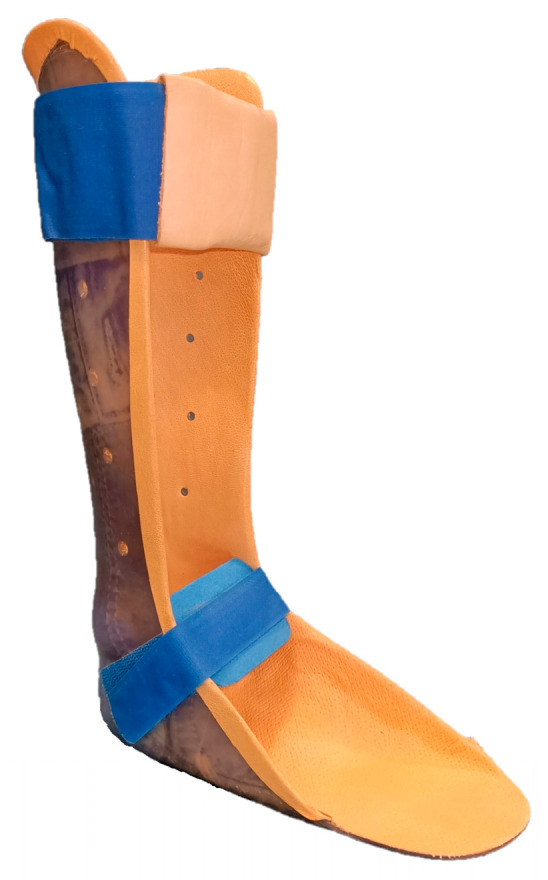
Example of SAFO used by the patients.

The SAFOs used by the patients were custom molded polypropylene AFOs (Figure 2) that had no joints and limited flexibility. They were designed to support the ankle during standing and during the stance phase of the gait and to prevent foot drop during swing. As for Botter, the stiffness and angle of the ankle at rest were adapted to each patient based on the experience of the technicians.

## 4. Assessments

All assessments were performed to monitor the orthoses functionality and disease progression. The assessments were performed with SAFO during the period of its utilization, with Botter after at least one month of its utilization and barefoot the same day of the Botter assessment. For excessive instability and unsafety referred by S1, barefoot analysis of the patient was only partially carried out. For both S1 and S2, the time between the SAFO and the Botter assessment did not exceed three months. For each condition, gait and postural stability were analyzed. S3 did not wear SAFO; therefore, only an evaluation of barefoot and with the bilateral Botter was performed.

Gait analysis was performed by means of a Vicon^®^ system (Oxford Metrics, Oxford, UK). The system was equipped with eight optoelectronic cameras (sampling frequency of 100 Hz), two force plates (AMTI, Watertown, MA, USA, sampling frequency of 1000 Hz) and two video cameras. A 10-m walkway allowed patients to reach and maintain a constant self-selected walking speed during acquisitions. Marker-set followed the Total3DGait protocol [20]. The adolescents walked until they effectively struck the force plates three times with each foot. No indications of striking the force plates were given to the patients. Kinematic and dynamic data were recorded. The motion of the markers underwent a Woltring filtering routine included in the Vicon^®^ software (Nexus, version 1.8). Gait cycles were identified automatically by the system when force plates were struck, otherwise manually. Averaged parameters of all the recorded gait cycles (at least six for each leg) were calculated.

The same force plates were used to measure the center of pressure (CoP) motion to evaluate standing stability.

The outcome measures were selected to explore five main aspects of AFO effectiveness: effect on push-off (primary outcome, that is, the expected result of Botter application); effect on deficiencies and compensations during gait (specific focus of AFOs for patients with HSMN; these aspects were studied and defined by Wojciechowski et al., 2017 [21]); general gait performance; standing stability; patient perspective.

The first aspect, i.e., push-off, should provide information on whether the children are capable to exploit the Botter carbon elasticity, since Botter is designed to increase push-off power. To answer this question, the following parameters were evaluated: maximum ankle push-off power; maximum ankle pre-swing moment; vertical ground reaction force (GRF) push-off peak. Ankle power was calculated as P = M × ω, where M is the moment of the ground reaction force with respect to the ankle center (midpoint between the two malleoli) and ω is the angular velocity of the foot relative to the shank. The calculation was performed with Aurion^®^ software (version 1.0, Milano, Italy) and based on the Total3DGait protocol. Since ankle movement was permitted by the AFOs only in the sagittal plane (relative to the shank), the power in this plane (around the medio-lateral axis) represented almost the entire power at the ankle.

Power generation at the ankle is a fundamental component of the propulsive push in the push-off phase [22,23,24], but it is not automatically transferred to a better global gait performance. Therefore, in order to evaluate the effects of these AFOs on a more general gait performance, stride length (normalized to height) and walking speed (self-selected, normalized to height) were considered.

More information on the global performance was obtained by functional tests (10 Meter Walk Test—10MTW; 2 Minute Walk Test—2MWT). Differently from the parameters identified by the gait analysis, that were calculated at a comfortable self-selected walking speed, the functional tests represented the best performance for speed (time in seconds to cover a 10 m distance for the 10MWT) and for endurance (distance in meters covered in 2 min).

The role of AFOs with the deficiencies was analyzed considering the following parameters:

- Maximum ankle dorsiflexion in the stance: inability to support the weight, mainly due to plantar-flexor weakness.
- Maximum plantarflexion in the swing: measurement of foot drop, determined by dorsal-flexor weakness.

The role of AFOs with the compensations was assessed by means of the following outcomes:

- Maximum knee flexion in the swing: compensation strategy to increase the clearance in the presence of foot drop.
- Maximum hip flexion in the swing: compensation strategy to increase the clearance in the presence of foot drop.
- Range of motion (ROM) of hip flexion in the terminal swing (measured as the difference between the maximum flexion in the swing and flexion at initial contact): represents the pass retract, that is, a quick extension of the hip before initial contact used to compensate for a toe-first landing in the presence of foot drop; this quick movement utilizes the inertia of the foot to obtain a passive dorsiflexion and, therefore, a better placement of the foot.
- Minimal knee flexion during the single support phase: measure of possible hyperextension of the knee to passively stabilize the knee itself and compensate for quadriceps weakness.
- Maximum power at the hip at the push-off: hip “pull-up”, that is, a compensation for a weak propulsion at the ankle level to flex the hip.
- Total ROM of pelvic rotation in the gait cycle (0–100%): rotation of the upper body to advance, used to compensate for a weak distal push-off power generation.

The aspect of standing stability was measured with CoP motion. For the calculation of the CoP, each patient was required to maintain a static upright posture on the force platform in the absence of external perturbations. The test was performed with eyes open and self-selected foot placement. It was indicated to remain still in position for an acquisition time of 30 s, after 5 s of initial adaptation (not recorded). In the literature, a recording time interval of 25–40 s is considered reliable: a test lasting 30 s is, therefore, considered adequate [25]. The Root Mean Square of CoP displacement (RMS) [26,27,28] and Mean CoP Velocity (MCV) [29,30,31] were calculated. MCV was found to correlate inversely with the dorsal-flexors strength and directly with the CMTES scale [32]. Before RMS and MCV calculations, the coordinates of the CoP were filtered with a moving average (span 50).

Finally, satisfaction in the use of orthoses was assessed through the Client Satisfaction with Device module of the Orthotics and Prosthetics Users questionnaire (CSD-OPUS) in its Italian version, validated by Bravini et al. (2014) [33]. CSD-OPUS consists of eight items which investigate the level of satisfaction with the orthosis. The patient is asked to express his degree of agreement/disagreement with each statement by choosing from four options: completely agree, agree, disagree, completely disagree. Each response is scored from 1 (strongly disagree) to 4 (strongly agree): the total score represents the position of the individual on the concept investigated, i.e., his appreciation for the orthosis.

## 5. Outcomes

**Figure 3:**
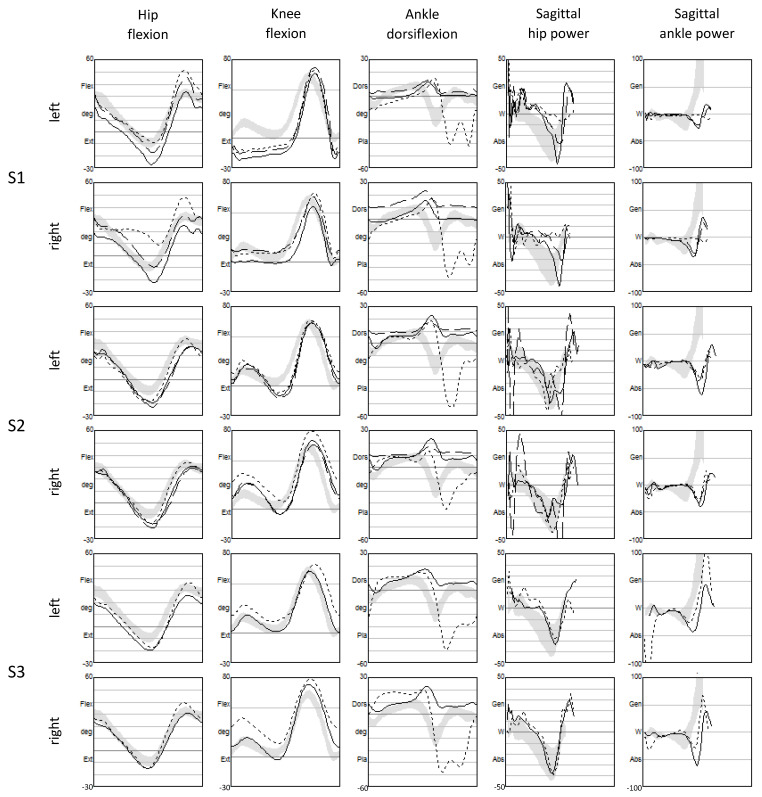
Sagittal kinematics and dynamics for the three subjects (S1, S2 and S3). Solid lines refer to the gait with Botter, dashed lines with SAFO and dotted lines barefoot. The gray bands represent the reference values (within standard deviations). They were calculated on a group of 20 healthy children acquired with the same protocol in the same laboratory.

Findings related to instrumental gait analysis acquired in the different conditions are presented in Figure 3 and Table 2. Only the most relevant changes are described below.

The maximum push-off power increased for S1 with both orthoses compared to the barefoot condition (from 1 W on the left and 5 W on the right to, respectively, 12 W/38 W with SAFO and 19 W/45 W with Botter). The changes in the maximum pre-swing ankle moment were: from 1 Nm/1 Nm barefoot to 5 Nm/6 Nm with SAFO and 4 Nm/6 Nm with Botter. Push-off was greater for S2 with Botter: 32 W/34 W versus 18 W/31 W barefoot and 19 W/20 W with SAFO. The maximum push-off power became more symmetrical for S3 with Botter: 52 W–52 W instead of 104 W–44 W with the barefoot condition.

Both SAFO and Botter improved stride length and self-selected speed in all the subjects.

The maximum plantarflexion during the swing (foot drop) was reduced with the orthoses: for S1 and S3, from around 50° barefoot to almost zero; for S2, from 54°/37° to −8°/−9° with SAFO and 5°/3° with Botter.

S1 could reduce the compensations of excessive hip flexion in the swing and of pass retract (hip terminal swing ROM) with both orthoses, especially for the right limb. Additionally, pelvis rotation decreased. Hip power at push-off (pull-on), instead, increased for the left limb. S2, with both orthoses, reduced the pass retract of the left limb. S3, with Botter, had a general reduction in compensations, especially regarding the range of rotation of the pelvis (decreased by about 25 degrees) and the increase in knee extension in single support (8 degrees on the left side and 17 on the right side).

Functional test measurements (10MWT, 2MWT) are reported in Table 3. S1 was not able to perform the 2MWT barefoot, but with SAFO covered a distance of 72 m in 2 min and with Botter, of 110 m. In the 10MWT, S1 needed 10.5 s to walk 10 m, while with both orthoses, was similarly faster (between 7 and 8 s). S2 increased the barefoot distance of the 2MWT from 130 m to 155 m with SAFO and to 170 m with Botter. The time of the 10MWT decreased both with SAFO and Botter from 7.6 s to around 6 s. S3 increased the 2MWT with Botter from 160 m to 210 m and improved the 10MWT from 6.9 s to 6.4 s.

The stabilometric analysis (COP sway and related parameters) is shown in Table 4. S1 was not able to stand alone for the requested 30 s. With SAFO and Botter, MCV and RMS were similar: respectively, 20 mm/s and 8 mm versus 18 mm/s and 6 mm. For S2, MCV and RMS were reduced by both orthoses, in particular, MCV decreased from 50 mm/s to 19 mm/s with SAFO and 21 mm/s with Botter. S3 had analogue results with and without Botter.

The CSD-OPUS questionnaire indicated better satisfaction with Botter both for S1 and S2. Out of a total of 32 points, Botter had a score of 24 with S1 and 29 with S2, while SAFO had 19 and 20, respectively. S3 rated Botter 23/32.

## 6. Discussion

This study aimed to evaluate the effect of dynamic (Botter) and solid AFOs on gait performance and standing stability in pediatric patients with HMSN. The expected results were better stability with both AFOs (compared to the barefoot condition) and better gait performance with Botter (compared to both the barefoot and solid AFO conditions).

The use of Botter allowed S1 and S2 to increase the power produced at the ankle, compared to both walking barefoot and with SAFO. S3, instead, had a more symmetrical ankle power production with respect to barefoot walking. The ankle dorsiflexion and plantarflexion were reduced compared to the condition without orthosis, as a direct consequence of the constraint introduced to limit the drop foot in the swing. However, the joint excursion of Botter was greater than that of the rigid orthosis: it was allowed by either a more open angle of the brace or the softness of the material. The possibility of exploiting a greater ROM at the ankle level could facilitate the residual muscular action of the plantar-flexors which, although deficient, were assisted and recruited as far as possible and not left completely at rest [19,34]. This, together with the elastic energy return of the carbon fibers, might allow the generation of a more effective push-off. The above is consistent with the increase in ankle power found by Dufek (2014) in a group of adult patients with HMSN thanks to the use of a custom-made carbon fiber brace [18]. Furthermore, our findings confirm that patients tend to undergo a reduction in maximum power at the ankle when using orthoses of greater rigidity [5,35,36]. The only case in which an increase did not occur was the left side of S3. Despite this reduction, however, a more symmetrical ankle push-off power was obtained. This could be more effective and functional over long distances. The symmetry is in fact related to energy efficiency [37].

We can define efficiency as the ratio between the distance traveled and the energy expended. An increase in efficiency with Botter, both with respect to the barefoot condition and with SAFO, is reasonably conceivable for all three patients thanks to the results of the 2MWT. This result is consistent with the findings of Bregman et al. (2012) in a different group of patients: the use of a carbon brace while walking reduced the metabolic cost by 9.8% compared to walking barefoot in adults with central neurological disorders (i.e., stroke and multiple sclerosis) [38]. The authors argued that this increased the maximum walking distance in patients with weak or easily fatigued plantar-flexors.

Considering the main compensations adopted in barefoot walking, such as increased hip and knee flexion in the swing (which defines the steppage gait), the pass retract and the increased power at the hip in the pre-swing, almost overlapping effects of Botter and SAFO may be assumed. Both showed an improvement (i.e., a reduction in compensations) compared to walking without an orthosis, with small differences between them. Therefore, we could speculate that the best outcome recorded at 2MWT with Botter compared to SAFO was not directly related to a reduction in compensatory movements, but to the improvement in push-off power and efficiency at the ankle (thanks to the carbon fiber).

The spatio-temporal parameters, in particular, speed and stride length, on the other hand, indicated an improvement in the use of orthoses compared to barefoot gait, but not a clear superiority of one brace over the other, given the variations found between the subjects. Even considering most of kinematics and dynamics parameters, the effects of the two orthoses were comparable over short distances (10MWT and gait analysis).

Regarding stability in a standing posture, as measured by means of MCV and RMS, an improvement was observed in S1 and S2 when using Botter and SAFO. However, S1 showed greater stability with Botter, while S2 with SAFO. S3, who was not assessed wearing SAFO, displayed a higher RMS with Botter than barefoot (12 mm vs. 9 mm). Looking at the last diagram in Table 4, it is possible to identify in the sway path two densification nuclei that could indicate a voluntary load transfer rather than a greater instability. However, this consideration is speculative and there are no objective measures to support it.

S1 and S2 expressed their views on both orthoses by filling in the CSD-OPUS satisfaction questionnaire. Both showed a higher level of satisfaction with Botter orthoses. In particular, both S1 and S2 reported that the carbon brace had a more acceptable weight, was more comfortable throughout the day and did not cause pain. Furthermore, the opinion expressed towards AFO Botter was lower than SAFO in none of the other aspects analyzed by the questionnaire. The only item for which Botter received negative opinions related to aesthetics (also in this case, however, to a lesser extent than SAFO).

This work has several limitations: the number of subjects investigated does not allow the generalization of the results; it has not been evaluated how a variation in the stiffness of both Botter and the solid AFOs could have influenced the performances; the impact of different levels of patient impairment has not been studied; the non-ecological environment may not reflect the actual behavior of the orthoses during daily life, for example, in different motor tasks (e.g., climbing stairs).

Future developments of this research will have to be performed on a larger sample, considering in detail the influence of both the characteristics of the subjects and those of the orthoses. These data will be able to define the optimal type of orthosis based on the patient’s needs.

## 7. Conclusions

Individually customized AFO may improve gait and postural balance while standing in adolescents affected by HMSN, with carbon AFO resulting more effective than SAFO.

Carbon AFO appears preferable to SAFO, according to the enquired adolescents.

## Figures and Tables

**Table 1:** Characteristics of the examined subjects (S1, S2 and S3). The muscle force, expressed in the Medical Research Council (MRC) scale (0 no contraction, 5 normal strength), and the ranges of motion suggest the degree of impairment of the various districts, providing an indication of the residual capacities and the necessary compensations.

		S1		S2		S3	
**Gender**		M		F		M	
**Age [years]**		14		14		14	
**Weight [kg]**		39.5		47		42	
**Height [cm]**		161		159		150	
**Passive range of motion**	Hip	preserved		preserved		preserved	
	Knee	with hyperextension (left > right)		preserved		with hyperextension (left > right)	
	Ankle	preserved		preserved		preserved	
**Muscle force** **[MRC scale]**		Left	Right	Left	Right	Left	Right
	Gluteus maximus	4	4	3	3	4	4
	Gluteus medius	3	3	3	3	4	4
	Adduttors	3	2	3	3	4	4
	Quadriceps	3	2	4	4	4	4
	Hamstrings	2	2	4	4	4	4
	Psoas	4	4	4	4	4	4
	Plantar-flexors	0	0	1	1	4	3
	Dorsal-flexors	0	0	1	1	2	1

**Table 2:** Results of instrumental gait analysis for the subjects S1, S2 and S3. The subjects were acquired barefoot (Bar), with SAFO and with Botter. Spatio-temporal parameters (STP) were normalized to subjects’ height (h). For each parameter, a reference value (Ref) is presented. Ref was calculated on a group of 20 healthy children acquired with the same protocol in the same laboratory. When available, left (L) and right (R) data are presented. Vertical Ground Reaction Force (GRF) is normalized to Body Weight (BW). Standard deviations are shown in brackets.

		S1						S2						S3				Ref
		Bar		SAFO		Botter		Bar		SAFO		Botter		Bar		Botter		
		L	R	L	R	L	R	L	R	L	R	L	R	L	R	L	R	
Push-off	Max Ankle push-off power [W]	1 (1)	5 (5)	12 (3)	38 (11)	19 (2)	45 (5)	18 (1)	31 (1)	19 (1)	20 (0)	32 (1)	34 (2)	104 (9)	44 (5)	52 (3)	52 (5)	84
	Max pre-swing Ankle moment [Nm]	1 (1)	1 (0)	5 (0)	6 (1)	4 (0)	6 (1)	2 (0)	3 (1)	5 (0)	5 (1)	3 (0)	3 (0)	9 (1)	5 (0)	9 (0)	6 (0)	8
	Vertical GRF push-off peak [%BW]	106 (0)	117 (4)	113 (4)	125 (6)	108 (2)	113 (4)	115 (1)	101 (3)	110 (1)	110 (2)	109 (3)	109 (0)	114 (1)	90 (4)	110 (3)	98 (3)	109
STP	Stride [%h]	58 (1)		78 (3)		79 (2)		56 (3)		76 (2)		69 (1)		68 (2)		75 (2)		85
	Speed [%h/s]	37 (1)		53 (5)		51 (2)		49 (4)		67 (2)		62 (1)		64 (3)		73 (3)		78
Deficits	Max stance Ankle dorsiflexion [deg]	18 (2)	17 (1)	14 (1)	25 (0)	13 (1)	17 (1)	20 (1)	14 (1)	18 (0)	17 (1)	23 (1)	24 (1)	16 (1)	22 (2)	18 (8)	25 (1)	8.6
	Max swing Ankle plantiflexion [deg]	44 (1)	49 (3)	−2 (0)	−10 (1)	2 (1)	2 (1)	54 (2)	37 (1)	−8 (1)	−9 (1)	5 (2)	3 (1)	52 (2)	50 (3)	−4 (2)	−2 (1)	−18
Compensations	Max Knee swing flexion [deg]	71 (2)	71 (12)	73 (3)	65 (4)	72 (1)	59 (1)	67 (3)	81 (3)	66 (3)	68 (1)	65 (2)	72 (2)	73 (2)	81 (1)	63 (2)	74 (1)	74
	Max Hip swing flexion [deg]	51 (2)	50 (5)	47 (3)	37 (3)	36 (1)	26 (3)	34 (1)	35 (2)	28 (2)	30 (2)	28 (3)	32 (2)	38 (1)	40 (1)	25 (2)	31 (1)	31
	Hip terminal swing ROM [deg]	20 (2)	18 (6)	19 (3)	8 (2)	17 (2)	8 (1)	18 (2)	9 (2)	5 (3)	4 (1)	4 (2)	4 (1)	15 (1)	13 (2)	7 (2)	9 (2)	9.0
	Min single support Knee flexion [deg]	−13 (1)	8 (0)	−14 (0)	9 (1)	−20 (1)	−1 (1)	−7 (0)	10 (9)	−13 (0)	−5 (1)	−10 (2)	−5 (2)	11 (4)	14 (3)	3 (3)	−3 (2)	−3
	Max Hip power at push-off [W]	5 (0)	22 (9)	26 (13)	19 (5)	26 (2)	18 (9)	34 (8)	32 (23)	46 (2)	45 (10)	27 (5)	27 (2)	19 (6)	33 (1)	29 (7)	35 (4)	35
	Pelvis rotation ROM [deg]	44 (2)	46 (2)	39 (1)	36 (4)	37 (4)	34 (1)	21 (7)	21 (2)	13 (3)	13 (4)	10 (1)	12 (2)	37 (1)	34 (1)	12 (2)	10 (2)	10

**Table 3:** Functional test results.

		Barefoot	SAFO	Botter
S1	2MWT [m]	NA	72	110
	10MWT [s]	10.5	7.8	7.3
S2	2MWT [m]	130	155	170
	10MWT [s]	7.6	6.0	6.2
S3	2MWT [m]	160	NA	210
	10MWT [s]	6.9	NA	6.4

**Table 4:** Stabilometric analysis. Sway path, Mean Cop Velocity (MCV) and Root Mean Square of CoP displacement (RMS) are shown.

		Barefoot	SAFO	Botter
S1			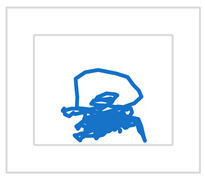	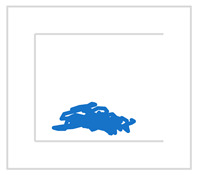
	MCV [mm/s]		20	18
	RMS [mm]		8	6
S2		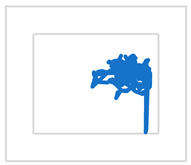	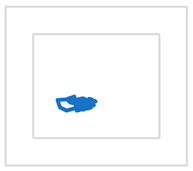	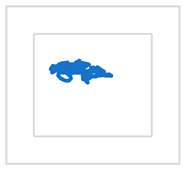
	MCV [mm/s]	50	19	21
	RMS [mm]	14	6	12
S3		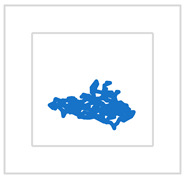		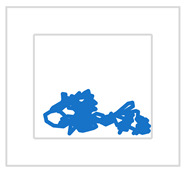
	MCV [mm/s]	20		23
	RMS [mm]	9		12

## Data Availability

The dataset analyzed during the current study is available from the corresponding author on reasonable request.
